# Resection of the Lower Jaw

**Published:** 1853-03

**Authors:** 

**Affiliations:** Hopkinsville, Ky.; Hopkinsville, Ky.


Art V.—*Resection of the Lower Jaw.* Successfully performed b Drs. Gaines & Henry, of Hopkinsville, Ky.

Elisha, a servant boy about 10 years of age, the property of Mrs. A. F. G., of Trigg County, was sent to us for medical treatment with a very prominent tumour occupying the greater portion of the left half of the inferior maxilla. The disease was what surgeon's term exostosis. The patient says that some 18 or 20 months ago whilst playing with his companions in a kitchen, one of them pushed him over, and in falling struck his jaw against the edge of an oven, which caused slight pain for the time being, but, that he has not suffered any since. When we first saw the boy, about 13 months since, the body of the lower-jaw on the left side, about midway between the angle and the symphysis begun to enlarge outwardly; this enlargement increased very slowly; the protruding tumor felt hard and smooth, but on pressure a slight crackling noise could be heard. Within the last six months, however, the growth has been much more rapid. On examination the tumor was found to be about the size of a goose egg, forming a solid mass apparently attached or forming part of the lower jaw on the left side, and extending from near the angle of the maxilla to the symphysis. The skin over the tumor does not appear to be altered in the slightest degree, but the swelling had projected much within the mouth, and jutted the teeth in against the tongue. It was evident that no remedial means could be of avail, except the complete removal of the diseased portion of the jaw, and as the tumor had evidently involved the greater portion of the latter, we determined to remove almost the left half of the lower maxilla from the angle to the symphysis. On the 12th of February the boy who had been previously prepared was placed upon the table and rendered insensible by chloroform which was administered by Dr. James Rodman, who, with other gentlemen of the medical faculty, kindly assisted in the operation. We commenced by extracting the left lower incisor tooth. An external incision was commenced just below the articulation and brought around under the jaw to the symphysis. A vertical incision was then made through the lip to the first incison. The next step was to secure the facial artery and vein, which was done by ligation above and below. The flap was now dissected up towards the eye and antrum, carefully avoiding injury to the parotid gland and duct. A straight saw was introduced and the bone clearly divided at the symphysis, then we passed a bistonry into the mouth and detached the soft parts from the bone a little beyond the angle. A chain saw was now passed under the bone and the bone divided just beyond the tumour. After a sufficient time had elapsed to afford an escape for whatever oozing might take place, the margins along the line of incision were accurately brought in opposition and secured by the interrupted and hare-lip sutures. The patient was now relieved from the effects of chloroform (under which he had been kept during the whole operation) by the admission of fresh air and the moderate use of stimulants, and was removed from the table in good condition.

We would take occasion to remark here, that had not this tumour, which created so much deformity, been removed, it would in a short time have worn out the patient.

We considered it most advisable to use the straight saw for dividing the bone at the symphysis, and the chain saw at the angle. Not being satisfied as to the exact character of the tumor, after its removal we proceeded to divide it longitudinally, and found it to be of the cystic kind, which was developed within the cancellated portion of the bone. We do not believe it to be of that character which is allied to cancer, either colloid or encephaloid. In some instances, where the diseases is circumscribed and does not involve both tables of the bone, a partial operation may with propriety be performed, but, in cases like the one under consideration, the operation must be more thorough in order to remove all the diseased bone. The patient has progressed very favorably. On the fifth day after the operation the sutures were removed, the line of incision having united by first intention. On the 10th day the patient left his bed, the wound being almost completely cicatrized, and now 18 days after the operation the patient is apparantly as well as ever. It is remarkable that so small an amount of deformity has resulted from the obliteration of so important a portion of the face. The void occasioned by insulating the bone and tumor has been completely filled by callus, and the divided extremities of the bone are firmly united by a strong ligamentous substance, which renders the jaw nearly as useful and symmetrical as before.

HOPKINSVILLE, MARCH 2nd, 1853.

